# Analyzing the Job Demands-Control-Support Model in Work-Life Balance: A Study among Nurses in the European Context

**DOI:** 10.3390/ijerph17082847

**Published:** 2020-04-21

**Authors:** Virginia Navajas-Romero, Antonio Ariza-Montes, Felipe Hernández-Perlines

**Affiliations:** 1 Department of Statistics, Management and Applied Economy. Universidad de Córdoba, 14014 Córdoba, Spain; 2 Management Departament, Universidad Loyola Andalucía, 14014 Córdoba, Spain; ariza@uloyola.es; 3 Departament of Business Administration; Universidad de Castilla La Mancha, 13071 Ciudad Real, Spain; felipe.HPerlines@uclm.es

**Keywords:** job demands, job control, social support, work–life balance, nurses

## Abstract

The balance of personal life with professional life is a topical issue that is increasingly worrisome due to globalization, the rapid introduction of new technologies into all areas of human life, the overlap between time between work and family, new organizational systems, and changes in the nature of work. This problem is accentuated by professions subjected to intense labor demands, as is the case of nurses. Adopting the Job Demand–Control–Support model, the main purpose of this research is to analyze how these factors lead to a greater or lesser degree of work–life balance. The research proposes a logistic regression model, which was constructed with a sample of 991 nursing professionals from the V European Working Conditions Survey. The results obtained confirm, on the one hand, that there is a significant effect of physical demands (but not psychological demands) on work–life balance. On the other hand, the moderating effects of job control are partially confirmed for psychological demands, and those of supervisor support (but not co-worker support) are partially confirmed for physical demands. In conclusion, the present research shows that effective management of nurses’ work context can decisively contribute to finding the difficult balance between personal and professional time.

## 1. Introduction

The balance of personal life with professional life is a topical issue that is increasingly worrisome due to globalization, the intrusion of new technologies into personal life, the overlap between work time and family time, new organizational systems, and changes in the nature of work [1]. This problem is accentuated in professions with intense working hours or schedules [2]. Nursing professionals are without a doubt in this situation. The role of a nurse is very demanding, as quick and effective responses are required to deal with the health needs of the community. Furthermore, it is an industry with an intensive work environment, which operates 24 h a day, 365 days a year, and sometimes requires exceeding the standard 40 h week of full-time employment for the production of services [3].

Work–life balance (WLB) is understood as several individual and structural constraints of their way of harmonizing work time and personal time according to an individual system of values, goals, and aspirations [4]. Although much research on the WLB has focused its analysis on the challenge of caring for children and the elderly [5,6,7], this is a topic of great importance in current academic research [8] because achieving an optimal WLB is critical to personal well-being [9,10]. Despite this, given the intense interconnectedness of work and personal time, many workers have serious difficulties defining the boundaries between them and often cross those boundaries, which has harmful consequences to their health and well-being [11]. Due to the changing nature of the current workforce, the WLB has a broader and higher perspective on the EU political agenda, encompassing aspects such as business organizational culture in terms of gender equality and social protection. In fact, at the Social Summit for Fair Employment and Growth held in Gothenburg in November 2017, the WLB was proclaimed as a European pillar of social rights [12]. Despite this, the peculiarities of the work environment that surrounds the nursing profession makes it difficult to reconcile work and family life, preventing a high level of work satisfaction from being achieved [13,14].

The job demand–control–support model (JDCS) [15,16,17,18] constitutes a very useful theoretical approach for understanding the characteristics of work and its consequences for the occupational health of employees [19]. In fact, this model has been used in many professions with the aim of studying a wide range of reactions that provoke tension in workers [20,21,22,23,24,25,26,27]. In particular, the JDC model establishes that factors such as the level of labor demands and the control the workers have over their work will affect the development of WLB. In subsequent model improvements, the level of perceived support at work was included as another relevant factor (the JDCS model).

Following this idea, the present study analyzes the effects caused by certain factors related to the context of work in nursing personnel (demands, control, and social support) and how they influence the WLB of these professionals. According to the JDCS model approach, the worst situation of WLB for workers would be manifested in those occupations characterized by high job demands, low control, and little social support. Previous studies with this model support the association between poor work results (for example, exhaustion, stress, poor well-being, etc.) with high levels of demands or work load (for example, [28,29,30]), low control over work, or the degree of autonomy in the application of labor decisions (for example, [31,32,33]). In addition, the low level of support perceived by colleagues or supervisors also influences (for example, [27,34]). The literature in general terms suggests that a low family–work balance is related to lower job satisfaction, higher turnover intentions, exhaustion, and deterioration of health [35,36], and a high family–work balance is related to higher levels of professional commitment and lower intentions to leave the profession [28,37,38]. However, although there are some studies that have tested the JDCS model with nursing personnel [28,39,40], the investigations that have related the model with the WLB are practically nonexistent.

Undoubtedly, nursing professionals carry out work of special interest to society, given the leading role of the health system in the welfare of any nation. Despite this, the work of nurses is characterized by high job demands: direct contact with patients, exhausting workdays, night shifts [41], and limited job control over the tasks they perform. This last circumstance is a result of the numerous regulations and operating protocols that regulate and limit the activity of these professionals, regulations that tend to be more rigorous when written by personnel outside the health care profession [42]. The other pillar on which the JDCS model is built is social support. Nursing requires much collaboration and teamwork. In this context, perceived social support is of great importance because, as Schwarzer and Knoll (2007) note, conflicts in work teams multiply when employees perceive little support from their supervisors and/or co-workers [43,44,45].

The consequences of the scenario described above are multifaceted: stress, alienation, low organizational commitment, and burnout. One of the least researched outcomes in academia from the perspective of the JDCS model is the problem that nurses have with balancing work time with personal time. Studies in other occupations suggest that the combination of high job demands and a great deal of autonomy can help reduce work–life conflict [46]. In contrast, individuals in professions that face many job demands within a context of low autonomy will have more difficulty achieving an adequate WLB [44]. Additionally, various studies suggest that supervisor and/or co-worker support contributes decisively to improving WLB [47].

Unlike other professionals, nurses offer an intangible service that is inextricably linked with the professional who performs it. Good management of work–family conflicts will improve the WLB of these professionals, which will translate into positive effects for the worker and the organization [48]. The purpose of this study is to contribute on the idiosyncrasies of the profession and the value the profession provides for citizens and the welfare state. The majority of studies on WLB adopt an approach based on worker health [49]. However, in the European context, there is little empirical research that analyzes both the main effects of the JDCS model and the multiplicative model in their relationship with WLB, which means that the impacts of job demands, job control, and social support have not received the attention they deserve in a profession so relevant in the European context [50]. Taking into account everything raised so far, this research seeks to (i) analyze job demands, job control, and social support in nursing staff, as well as assess whether these factors lead to a greater or lesser degree of WLB in European hospitals; and (ii) explore whether these models differ from those identified in the previous literature. The following research question is derived from these objectives: Does this organizational model allow its workers to have a high WLB according to the JDCS model that relates labor demands, labor control, and social support?

## 2. Managing Demands, Control and Support in an Organizational Context in Nursing

Health is one of the fundamental pillars of a welfare society. The World Health Organization estimates that the health care sector workforce in 2013 included 20.7 million nurses out of a total of 43.5 million health workers worldwide [51,52]. The well-being of workers is essential for the health of society [49]. Balancing work and family life in the nursing sector to achieve a high degree of well-being is crucial for the development of the service [53], since well-being impacts the quality of patient care [54], with poor well-being increasing the risk of negligence and/or abuse [55].

The sector is characterized by high job demands, both physical and psychological. The situation with respect the physical demands of nurse workers reflects the need to work with limited resources, both in terms of personnel and equipment [56], which causes a level of emotional strain that approaches exhaustion [52,53]. The nursing sector is the sector with the highest rate of nonfatal occupational diseases and injuries, which causes an increase in sick leave [57]. Factors such as stress, poor health, and depression negatively affect WLB [58,59]. Regarding the psychological demands, nurses must make urgent and critical decisions that involve a vital risk for the patient [60]; thus, nursing professionals are continuously exposed to traumatic events [61]. Health care services are needed 24 h a day, seven days a week. This need may contribute to greater job stability in health care than in other sectors [62], but it also implies an internal organization based on exhausting workloads and 12 h continuous work shifts, which hinder the conciliation of work with personal and family life [63].

From the perspective of job control, it deepens knowledge of the skill discretion and the decision authority of this sector, and nursing work requires multiple skills [64]. According to Ulrich, Barden, Cassidy, and Varn-Davis, there are different skills that workers in this sector have to have, such as communication, collaboration, and resource management and leadership skills [65]. At the same time, nursing professionals usually have little autonomy in carrying out their work activities [66], which greatly limits their decision-making capacity [67]. However, nurses who have higher levels of work autonomy tend to be high-performing, satisfied, and committed workers [68]. With respect to social support in health, Olmedo (2009) reports the presence of a complex web of interpersonal relationships with conflicting power dynamics between physicians (who are mostly male) and nurses, whose profession is still highly feminized [69,70]. Work pressures, limited control, and the complex social system trigger deterioration of the work environment [71], resulting in absenteeism, high turnover, depression, and even suicide [72]. Nurses who consider these conditions unsustainable eventually abandon the profession in search of jobs with fewer demands and better working conditions that will improve their personal quality of life, including the balance between work and family time [73].

Although some previous studies have applied the JDCS model in the nursing sector, they have generally done so from analytical approaches and perspectives that differ from those presented in this article. From the perspective of job demands, Heijden et al. (2018) confirm that job demands are positively correlated with job turnover, while the availability of resources and the experience of nurses are inversely related to turnover [74]. At the end of the last century, De Jonge et al. (1999) claimed that the combination of job demands and job control can predict the health of employees [75,76]. From the perspective of job control, the work of Yamaguchi et al. (2016) confirms that the absence of job control increases the likelihood that nursing personnel will abandon nursing as a career [77]. More recently, Mark and Smith (2012) have added that rewards, decision-making skills, and skill discretion decrease depression and anxiety among these professionals, which is often motivated by a lack of resources and a lack of time to attend to patients [78,79]. Finally, from social support perspective, Sigurdardottir et al. (2015) show that training programs improve nursing professionals’ perceptions of social support (from both supervisors and co-workers) [80,81]. Stress is another topic of interest analyzed with this model. Thus, Nabirye et al. (2011) note that the role conflict and ambiguity that characterize the work of more experienced nurses lead to increased stress [82,83].

The effects that such a demanding profession exerts on health and well-being have also been the subject of analysis in academia [84]. The study of Xanthopoulou et al. (2009) provides key evidence of pathways leading to the well-being of nursing personnel in general hospitals [85]. Another study using the JDCS model in Uganda concluded that younger nurses are more satisfied than older nurses [82]. These authors suggest that with age, family responsibilities increase, and consequently, the likelihood of conflicts between personal and professional life increases. Other authors focus on analyzing the most important antecedents of the psychosocial well-being of nurses, highlighting the importance of social support to improve job satisfaction and stress [86]. Following this idea, two studies carried out in Sweden show the positive effect that empowerment [87] and social support [88] exert against burnout in nursing personnel.

## 3. Research Hypotheses

The three previously described job strain variables should have an interactive effect on the WLB of nurses. Additionally, the interaction among these variables can reduce the relationship between job demands and employees’ WLB. Against the reference framework described in the previous paragraphs and based on the literature review, the following research hypotheses are proposed:

**Hypothesis 1.**  *The high job demands faced by nursing professionals will be negatively associated with work−life balance.*

**Hypothesis 2.**  *Job control will reduce the effects of job demands on work−life balance.*

**Hypothesis 3.**  *Supervisors/co-workers will reduce the effects of job demands on work−life balance.*

**Figure 1:**
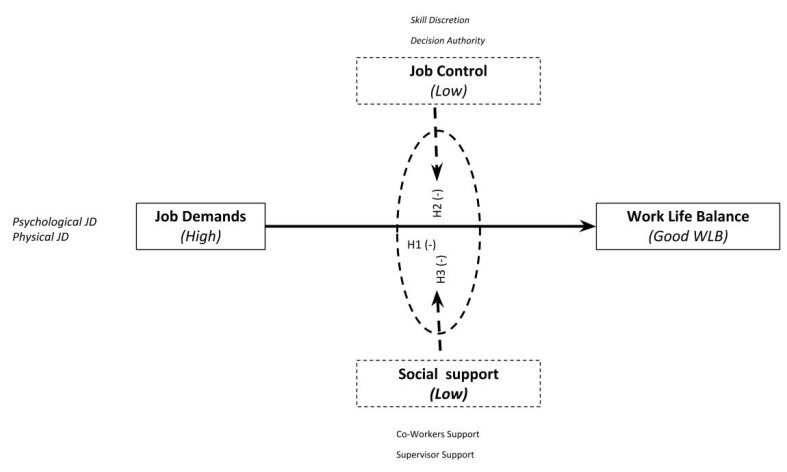
Research model. Source: Prepared by the authors.

The following Figure 1 summarizes the theoretical model and research hypotheses.

## 4. Methodology

### 4.1. Data Collection and Sampling

For the development of this research, information collected by the European Foundation for the Improvement of Living and Working Conditions regarding the work environment and working conditions in 35 European countries was used (EU28, Norway, Switzerland, Albania, the former Yugoslav Republic of Macedonia, Montenegro, Serbia, and Turkey during the period from February to September 2015. The European Working Conditions Survey (EWCS) (The complete questionnaire can be found in the European Foundation for the Improvement of Living and Working Conditions (2019). See the following link: https://www.eurofound.europa.eu/sites/default/files/page/field_ef_documents/6th_ewcs_2015_final_source_master_questionnaire.pdf) is conducted every five years based on a questionnaire-based interview. A subsample of 991 nursing professionals (ISCO Code 222) (Classification structure of the International Labor Organization (ILO) that organizes jobs according to tasks and functions. Specifically, group 222 includes “Nursing and midwifery professionals”) was extracted to achieve the objectives of this study. The majority of the respondents were women (89.1% of the total), with an average age of 42.8 years (SD = 11.0).

### 4.2. Measures and Methodology: The Binary Logistic Regression Model

The dependent variable of this study was WLB, measured by an indicator composed of four items that ask the following: (a) How are your working time arrangements set? (b) How do your working hours fit in with your family or social commitments outside work? (c) How often have you worked during your free time to meet work demands? (d) Would you say that arranging to take an hour or two off during working hours to take care of personal or family matters is easy or not? The Cronbach’s α of the four-item scale was 0.898. Subjects with good WLB were coded as 1, while those with a lower level of WLB were coded as 0.

As indicated by Rugulies et al. (2010), the Copenhagen Psychosocial Questionnaire is considered a very effective tool for assessing the psychosocial work environment [89]. Different authors, such as Widerszal-Bazyl (2017), have demonstrated the psychometric properties of this questionnaire [90]. The extensive information provided by the European Working Conditions Survey permits the rigorous reproduction of the different scales included in the Copenhagen Psychosocial Questionnaire [9]. To meet the objectives of the present study, five indexes related to job demands, work organization, relationships, leadership, etc. were calculated. Twenty-three items from the EWCS were used to generate five indexes: (1) Psychological Job Demands (Cronbach’s α = 0.736); (2) Skill Discretion (Cronbach’s α = 0.695); (3) Decision Authority (Cronbach’s α = 0.770); (4) Co-worker Support (only one item); and (5) Supervisor Support (Cronbach’s α = 0.905).

Finally, physical job demands were measured with the Job Content Questionnaire (JCQ). This scale evaluates the social and psychological characteristics of a particular job. Some examples of the 13 items used to measure the risk of physical job demands are the following: “Are you exposed at work to high temperatures that make you perspire even when you are not working?”, “Do you handle or have skin contact with chemical products or substances?” or “Does your main job involve lifting or moving people?” Cronbach’s Alpha for this scale was 0.772.

The statistical analysis applied in this study was the binary logistic regression model. This method is one of the most frequently applied statistical approaches for developing social prediction models with binary results [91,92]. Logistic regression has been used with the Karasek model for example [93,94,95] and specifically in the field of nursing (for example, [96,97,98]). This method allows the determination of the probability that a certain event—good balance between personal and professional life—will occur compared with the probability that the opposite event will occur. In this study, the Hosmer−Lemeshow goodness of fit test was used as a measure of overall validity, and the Wald test was used for the analysis of individual variables.

## 5. Results

In the initial estimate of the central variable of this study, the general sample of the nursing professionals appears to present some problems balancing their personal time and work time, with an average score of only 0.5071 on a scale of 0 (poor WLB) to 1 (good WLB). In addition, this problem seems slightly more pressing among women (0.5067) than among men (0.5102).

In relation to the demands to which nursing professionals are subjected, it must be emphasized that the weight of psychological demands (0.3513) is, in all cases, greater than that of physical demands (0.2384). Additionally, women perceive more demands than men, both physical (0.2387 vs. 0.2364) and psychological (0.3524 vs. 0.3421).

Nursing professionals have greater skill discretion (0.6607) than decision authority (0.5698). This general trend does not differ by gender, although male nurses have greater skill discretion than female nurses (0.6667 vs. 0.6600), while female nurses have greater decision authority than male nurses (0.5714 vs. 0.5563).

Finally, the perceived supervisor support (0.2564) is much higher than the perceived support from co-workers (0.1893), a circumstance that should be the subject of analysis and deep debate within the profession. In addition, male nurses have more social support mechanisms than female nurses in regard to support from both supervisors (0.2617 vs. 0.2558) and co-workers (0.1952 vs. 0.1886).

### 5.1. Job Demand−Control−Support Factors and Work-Life Balance

The individual relationships between the different factors of the JDCS model and WLB are presented in Table 1. First, it should be noted that the fundamental principles of the JDCS model are confirmed. The high job demands, the low job control, and the low social support are negatively correlated with the outcome variable, i.e., the WLB indicator. However, these results must be taken with caution because the only variables that are statistically significant are those related to the physical (β = −0.613, *p* = 0.000) and psychological demands (β = −0.520, *p* = 0.001) on the nursing professionals. In fact, these two scales have the strongest relationship with the outcomes variable, followed by the two scales of social support and, finally, the measures of job control.

An examination of each of the individual components shows that physical demands are more important than psychological demands for predicting WLB among nursing and professionals. In addition, the association between “low decision authority” (β = −0.188, *p* = 0.250) and WLB is more intense than the association between “low skill discretion” (β = −0.087, *p* = 0.555) and WLB. Likewise, the influence of supervisor support on WLB balance (β = −0.412, *p* = 0.178) is greater than the association between support from co-workers and WLB (β = −0.257, *p* = 0.260). 

### 5.2. Strain Model

The original proposal of the Karasek model (1979) is presented in Table 2, which shows the analysis of the association between WLB and job demands (physical and psychological) and job control (skill discretion and decision authority) and the interaction between both dimensions [16].

The results show a direct negative relationship between physical job demands and WLB (β = −0.397, *p* < 0.05). These results partially confirm Hypothesis 1.

Additionally, to test for interaction effects, interaction terms were introduced in the logistic regression model. Table 2 shows that the effect of psychological job demands on WLB is moderated when skill discretion is involved (β = −0.533, *p* < 0.01). This means that control over work (discretion to solve problems on your own and performing varied activities for which it is necessary to learn new skills) enriches nurses’ work and possibly their personal life, cushioning the negative effect that the psychological demands of the profession exert on WLB. In other words, in the presence of low job control, the negative effect of psychological demands on WLB is more intense (β = −0.533, Sig. 0.006) than when greater job control is present (β = −0.520, Sig. 0.001). These results partially support Hypothesis 2 because the moderating effect of decision authority in the job demands−WLB relationship does not obtain statistically significant results for physical demands (β = 0.007, Sig. 0.983) or for psychological demands (β = −0.302, Sig. 0.335).

### 5.3. Iso-Strain Model

Subsequently, variables related to social support were incorporated into the model to contrast the hypotheses of the iso-strain model. Table 3 shows the adjusted association between WLB and job demands (physical and psychological), job control (skill discretion and decision authority), and social support (co-workers and supervisors) and their respective interactions.

The new model presents some novelties regarding the formulated hypotheses. The most notable results are as follows. First, the direct relationship of job demands with WLB disappears from the overall model. The association of physical and psychological demands with WLB occurs in interaction with variables related to job control and social support. Thus, Table 3 shows that the strain-by-control interaction term at the 0.05 level only manifests when decision authority is present. This variable exerts a moderating effect on the psychological job demands−WLB relationship (β = −0.621, *p* < 0.05). This result partially confirms research hypothesis 2 as it indicates that decision authority, but not skill discretion, has a modulating effect.

Finally, the strain-by-support interaction term at the 0.05 level was analyzed. Table 3 shows that supervisor support moderates the effect of physical demands on WLB (β = −0.749, *p* < 0.01). This result partially confirms research hypothesis 3 as it indicates that supervisor support, but not co-worker support, has a moderating effect.

## 6. Discussion

The main objective of the present study was to test the efficacy of the JDCS model for predicting WLB among nursing professionals. Nursing presents its own particularities and is critical for maintaining the welfare state in Europe; however, nursing care takes place in one of the most stressful work environments that exists [96]. As noted by Karatepe and Uludag (2007), the health care sector is unique due to its high level of stress and its intense physical, psychological, and emotional demands [99].

Some studies, such as that of Ghislieri et al. (2017) performed with a sample of 500 nurses working in an Italian hospital, confirm that nurses have difficulties balancing work time and personal time [100]. To examine this issue among nursing professionals, the current study adopts the JDCS model as a reference framework, developing several logistic regression models that attempt to explain the direct and interactive effects of three fundamental variables: job demands, job control, and social support. The results obtained from a large sample of 991 European nursing professionals partially confirm both the strain hypothesis and the iso-strain hypothesis of the Johnson and Hall (1988) and Karasek (1979) models [16,17].

First, with respect to Hypothesis 1, a significant effect of physical (but not psychological) demands on WLB is confirmed. The physical demands that most affect nurses are, in the following order, handling or being in direct contact with materials that can be infectious, such as waste, bodily fluids, laboratory materials, etc.; lifting or moving people; having to maintain tiring or painful positions; performing repetitive hand or arm movement; handling or being in skin contact with chemical products or substances; and carrying or moving heavy loads. Being subjected to such physical demands causes nurses to feel that their work does not fit well with family or social commitments. The high physical demands cause a decrease in the well-being of workers linked to the work–family conflict because it includes aspects of physical fatigue, pain, and insecurity in the work environment [101], reducing the physical resources that a worker has to their WLB [47]. According to the World Health Organization, poor well-being in the workplace is one of the most important causes of absenteeism, turnover, and poor performance in the workplace [51]. This result is consistent with the study by Hussain et al. (2012), who highlight the high job demands that nurses face as well as the very poor working conditions, which translate into serious difficulties reconciling work and family time [102]. These authors warn of the risk that nurses will abandon the profession in search of less demanding and stressful options. The physical and psychological demands that nursing professionals face are well known and are overwhelmingly related to their close interactions with patients [103,104]. The results of our study indicate that nurses experience greater strain from physical demands than from psychological demands, perhaps because, as Qureshi (2018) warns, direct contact with patients requires high physical effort to lift or move people or heavy objects, maintain tiring or uncomfortable positions, and perform repetitive tasks or movements with one’s hands or arms [105]. Similarly, Greenglass et al. (2001) and Garrett and McDaniel (2001) suggest that high physical demands may originate from the excessive workload and atypical schedules that include shift work, weekends, and nights [106,107,108,109]. These factors cause an imbalance between professional and personal demands, which manifests as lower organizational commitment, exhaustion, work stress, dissatisfaction, and, directly related to the object of this research, a worse personal–professional life balance [110].

Hypothesis 2 of this research was examined in a second phase intended to demonstrate the modulating effect of job control on WLB. The results of this study confirm this moderating effect in the case of psychological demands, which suggests that increasing the decision-making capacity of nursing professionals will improve their mood, vitality, and general interest, thus cushioning the direct effect that the demanding work exerts on stress, overwork, and personal life–professional life conflict [111]. Therefore, this research empirically confirms that being able choose the order in which one will perform tasks, being able to work at one’s own pace and using one’s own methods, being consulted about objectives that affect one’s work, being able to influence the decisions that are important, or having the opportunity to apply one’s own ideas at work, among other discretionary acts, cushions the negative effect that the psychological pressure of nursing work exerts on WLB. These results are in line with the study by Pisarski et al. (2006), who show that increased control in the workplace generates better WLB, especially when the individual is able to control his or her work hours and/or experiences an increase in schedule flexibility [112,113,114,115]. For many health professionals, their commitment to their work and professional career is a priority that takes them away from family and social relationships and causes them to dedicate less time than they would like to family responsibilities [116]. This extreme and rigid dedication causes a high level of exhaustion and a sensation of lethargy and depersonalization [117]. The debate on the effects of this professional dedication on WLB as one’s health care career advances has a long history, but the issue seems far from a closed topic [118]. Decades ago, Hirschman (1970) reported the need to introduce changes in hospital work practices to correct this problem [119]. More recently, Mushfiqur et al. (2018) emphasize the moral obligation to change matters related to the management of health institutions, including work hours and locations, the relationship with the environment, and, of course, better balance between personal life and professional life [120]. Increasing the flexibility of work schedules is one of the most common strategies to mitigate this problem. Thus, the Medical Women’s Federation (MWF) (2018) and Adisa et al. (2017) promote part-time work as a way to effectively improve WLB and create a positive impact on both Social Security and the lives of workers [118,119,120,121].

Finally, Hypothesis 3 of this research aimed to verify the moderating effect of social support in the job demand–WLB relationship. This moderating effect is partially confirmed for physical demands in the presence of supervisor support; that is, the negative effect of physical demands on WLB is mitigated when supervisor support is available. In conclusion, the physical demands (handling potentially infectious materials, moving patients, maintaining painful positions) that nursing professionals face generate problems with WLB, but these problems are reduced if nurses have the support of their immediate supervisors. A similar conclusion was reached by Abdul-Rashid et al. (2017), who applied a structural equations model in a sample of nurses from public hospitals in Malaysia [122]. Social support is decisive in an occupation in which teamwork, specifically cooperative work, occupies a leading role [123,124]. The creation of a work environment based on open communication and social support from co-workers and supervisors necessarily increases WLB [125]. Professionals in nursing units face complex problems that require very specific skills, support, and a great capacity for adaptation [126]. In this sense, support from both co-workers and supervisors can moderate the harmful effects of nursing demands on WLB. The studies of Tucker et al. (2018), Somers et al. (2018), and Jennings (2007) confirm that positive interpersonal relationships with supervisors improve safety, mutual respect, and positive feelings, which translate to greater WLB [127,128,129] The results of our research support the cushioning effect of social support (at least in terms of supervisor support) in the job demands–WLB relationship, thus supporting Hypothesis 3.

## 7. Conclusions and Limitations

This study yields important theoretical and practical implications for the nursing profession in Europe. At the theoretical level, understanding how the JDCS model works in a sector such as nursing, where complex and dynamic tasks are performed, can decisively contribute to improving the WLB of professionals subjected to intense work demands. From a practical perspective, the findings of this study corroborate the idea that job demands are not the only variables that affect WLB, particularly when employees perceive job control and/or social support from their organizations. Consequently, the industry must analyze the workplace factors that affect WLB. Human resource managers should explore new tools to provide employees with control over their daily activities, especially in an occupation such as nursing, which involves direct contact with patients and in which the quality of the services provided is conditioned by the workers’ decision-making capacity and freedom of actions. All progress in this regard (for example, modifying action protocols) will lead to greater autonomy, which should translate into better WLB. In addition, managers should promote a cooperative work environment based on an organizational culture of support, and teamwork should be encouraged. Investing in the training of work teams will improve aspects such as the organizational climate and social support, which will translate into improvements within the organization (for example, in the organization of work shifts) and in the attitude towards patient service. These strategies should reduce job stress and, as a result, increase the WLB of workers.

As is often the case in empirical research conducted in the field of social sciences, the results obtained should be interpreted with caution. First, a causal relationship between variables cannot be established since this is a cross-sectional study. Second, the study of the JDCS model is based on self-assessed measures and is therefore susceptible to bias; however, Pelfrene et al. (2002) corroborated that studies based on self-assessed measures support the strain hypothesis of the model to a greater extent than studies that use more objective evaluations [130]. Third, the research was developed in a specific socio-geographic scenario (Europe) where very different health systems and labor codes coexist that condition the fundamental variables of the JDCS model. Fourth, no control variables have been introduced, despite being a highly feminized profession. Therefore, it would not be prudent to generalize these assumptions and ideas to other work environments. Future studies should investigate other groups in the health industry (for example, doctors or hospital managers) and analyze the influence of different geographic areas to allow comparisons among different cultural environments.

## Figures and Tables

**Table 1:** Logistic regression: factors that determine work−life balance (high/low score).

Variables in the Model					Odds Ratios		
					95% C.I. for OR		
Variables	B	Standard	Wald	*p*	OR	Lower	Upper
**Job Demands**							
Psychological JD (high)	−0.520	0.151	11.863	0.001	0.594	0.442	0.799
Physical JD (high)	−0.613	0.15	16.711	0.000	0.542	0.404	0.727
**Job Control**							
Skill discretion (low)	−0.087	0.148	0.348	0.555	0.917	0.686	1.224
Decision authority (low)	−0.188	0.163	1.324	0.250	0.829	0.602	1.141
**Social Support**							
Co-worker support (low)	−0.257	0.228	1.271	0.260	0.773	0.494	1.209
Supervisor support (low)	0.412	0.306	1.811	0.178	0.662	0.364	1.207

**Table 2:** Adjusted association between job demands, decision latitude, their interaction, and work−life balance index (Strain Model).

Variables in the Model					Odds Ratios		
					95% C.I. for OR		
Variables	B	Standard	Wald	*p*	OR	Lower	Upper
**Job Demands**							
Psychological JD (high)	0.025	0.321	0.006	0.937	1.026	0.546	1.926
Physical JD (high)	−0.397	0.193	4.223	0.040	0.672	0.460	0.982
**Job Control**							
Skill discretion (low)	0.320	0.248	1.673	0.196	1.377	0.848	2.237
Decision authority (low)	−0.095	0.216	0.192	0.661	0.917	0.596	1.389
**Moderator effect of JC**							
PhyJD x JC (Skill)	−0.257	0.329	0.541	0.462	0.785	0.412	1.495
PhyJD x JC (Dauth)	0.007	0.320	0.000	0.983	1.007	0.538	1.886
PsyJD x JC (Skill)	−0.533	0.195	7.467	0.006	0.587	0.400	0.860
PsyJD x JC (Skill)	−0.302	0.313	0.929	0.335	0.739	0.400	1.366

**Table 3:** Adjusted association between job demands, decision latitude, social support, and their interaction and work−life balance index (iso-strain model).

Variables in the Model					Odds Ratios		
					95% C.I. for OR		
Variables	B	Standard	Wald	*p*	OR	Lower	Upper
**Job Demands**							
Psychological JD (high)	−0.122	0.466	0.069	0.793	0.885	0.355	2.206
Physical JD (high)	−0.397	0.193	4.223	0.040	0.672	0.460	0.982
**Job Control**							
Skill discretion (low)	−0.041	0.351	0.014	0.906	0.960	0.483	1.908
Decision authority (low)	−0.175	0.301	0.337	0.562	0.840	0.466	1.515
**Moderator effect of JC**							
PhyJD x JC (Skill)	0.259	0.485	0.285	0.594	1.295	0.500	3.354
PhyJD x JC (Dauth)	−0.341	0.461	0.548	0.459	0.711	0.288	1.754
PsyJD x JC (Skill)	−0.348	0.487	0.511	0.475	0.706	0.272	1.834
PsyJD x JC (Skill)	−0.621	0.264	5.543	0.019	0.538	0.321	0.901
**Social Support**							
Co-worker support (low)	0.029	0.466	0.004	0.950	1.03	0.413	2.566
Supervisor support (low)	−0.215	0.485	0.196	0.658	0.807	0.312	2.087
**Moderator effect of Social Support**							
PhyJD x JC (Skill)	0.769	0.521	2.178	0.140	2.157	0.777	5.989
PhyJD x JC (Dauth)	−0.749	0.267	7.848	0.005	0.473	0.280	0.799
PsyJD x JC (Skill)	−0.814	0.519	2.457	1.117	0.443	0.160	1.226
PsyJD x JC (Skill)	0.522	0.492	1.126	0.289	1.686	0.642	4.423
